# How Do Humans Control Physiological Strain during Strenuous Endurance Exercise?

**DOI:** 10.1371/journal.pone.0002943

**Published:** 2008-08-13

**Authors:** Jonathan Esteve-Lanao, Alejandro Lucia, Jos J. deKoning, Carl Foster

**Affiliations:** 1 Department of Exercise Physiology, European University of Madrid, Madrid, Spain; 2 Faculty MOVE, VU University-Amsterdam, Amsterdam, the Netherlands; 3 Department of Exercise and Sport Sciences, University of Wisconsin-La Crosse, LaCrosse, Wisconsin, United States of America; Pennington Biomedical Research Center, United States of America

## Abstract

**Background:**

Distance running performance is a viable model of human locomotion.

**Methodology/Principal Findings:**

To evaluate the physiologic strain during competitions ranging from 5–100 km, we evaluated heart rate (HR) records of competitive runners (n = 211). We found evidence that: 1) physiologic strain (% of maximum HR (%HRmax)) increased in proportional manner relative to distance completed, and was regulated by variations in running pace; 2) the %HRmax achieved decreased with relative distance; 3) slower runners had similar %HRmax response within a racing distance compared to faster runners, and despite differences in pace, the profile of %HRmax during a race was very similar in runners of differing ability; and 4) in cases where there was a discontinuity in the running performance, there was evidence that physiologic effort was maintained for some time even after the pace had decreased.

**Conclusions/Significance:**

The overall results suggest that athletes are actively regulating their relative physiologic strain during competition, although there is evidence of poor regulation in the case of competitive failures.

## Introduction

The image of the long distance runner evokes the popular fantasy of extraordinary effort. Middle and long distance running competition represents the greatest degree and limitations of human locomotive endurance capacity. In an attempt to regulate running effort, various physiological systems interact to preserve homeostasis for the period of time associated with the event [1]. Beyond the contemporary world of sports performance, there is evidence to suggest that locomotion over long distances may have allowed early humans to exploit a unique evolutionary niche as “persistence (-tant) hunters” [2]. Although we know much about the biological characteristics of humans who are elite runners [3]–[4], and how they differ from less accomplished runners [5]–[7], we know comparatively less about what actually transpires during running competition [8]–[13]. Further, the results from research on other sports involving less ‘natural’ types of locomotion for humans (cycling, skating) [14]–[24] may not necessarily be extrapolated to the natural bipedal motion that humans have adopted over centuries of evolution.

Within the last 10 years a number of studies focusing on pacing strategy have revealed a pattern of response during actual [14], [15], [19]–[24] and/or simulated [16]–[18] competitions in cycling/skating events ranging from ∼30 seconds to 3 weeks. An hypothesis arising during this same period suggests that that humans regulate their effort during competition based on the *anticipation* or estimation of when the exercise will end. The “central nervous system (CNS) governor” (i.e., central governor) is purported to provide feedback from a variety of receptors that monitor the physiological response to the demands of the activity in order to preserve internal homeostasis [24]–[26]. Recent data from our laboratory [14], [19], [27] and elsewhere [28] seem to support, at least partly, the hypothesis of a pre-existing template or plan for an event which is based on either practice or prior competitive experience as reflected by the fact that simple physiological (heart rate, HR) or psycho-physiological markers (rating of perceived exertion, RPE) is scaled to competitive efforts in such a way that the progressive development of fatigue is proportional to the relative percentage of the event completed. During repeated-sprint (‘all out’) exercise, however, the aforementioned *anticipated* regulation coming from the CNS does not occur [29]. Further, other studies show that humans adjust muscle power output during simulated endurance competitions depending mainly on sensory feed-back derived from progressively fatiguing muscles irrespective of their previous competitive experience [30]. This sensory feed-back is obtained principally from inhibitory information to the CNS based on the O_2_-dependent accumulation of metabolic byproducts in the working locomotor muscles that constantly modulates central motor output to muscles [31]. Thus, the rate of peripheral fatigue development is highly regulated [32] and constant sensory feed-back from working muscles to the CNS prevents muscle peripheral fatigue from surpassing a dangerous level or ‘threshold’ [33] leading to potentially harmful consequences ranging from simple muscle task failure to muscle structural damage [34].

Since there is reasonable evidence suggesting that we may be approaching the limit for physiological capacity [35], [36], interest in how humans expend limited energetic resources during vigorous exertion of varying duration is an evolving area of research interest. To increase the understanding of how humans regulate their responses during strenuous running exercise we observed the spontaneous variations of running pace (e.g. net effective muscular power output) and HR (a widely used index of physiologic strain) in a large number of competitive runners during a variety of competitive running events, ranging from relatively short (5 km) to very long distances (100 km).

The primary aim of our study is to examine the HR response, and thus exercise intensity, variance in relation to race distance. Specifically, we hypothesize that HR would increase in a manner scaled to the proportional running distance of the event. A secondary aim of our investigation was to assess if HR response over the different distances varies with individual running ability. Thus, we hypothesize that adept and less adept runners would display similar physiologic strain that would be proportional to the duration of their event. Our final aim was to examine the HR response of those runners showing clear discontinuities in performance within a given race characterized by an abrupt decrease in running velocity. We hypothesize that those performances showing discontinuity (e.g., “hitting the wall” in the marathon) would be characterized by evidence of poor physiologic regulation.

## Methods

### Subjects

To examine our hypothesis, we examined 211 male middle and long distance runners [mean±SEM (range) age: 32±8 years [20], [45]] of various abilities. All trained for and entered competitions with the intent of achieving their best possible performances. Although not elite performers, all were serious competitors and some were successful in regional competitions. Most runners competed in several different types of events during various portions of their training cycle. Laboratory tests and heart rate (HR) recordings (see below) were assessed as a normal function of controlling training and racing by their coach. In this study, subjects were not exposed to experimental procedures or laboratory methods that they would not have performed for non-investigational reasons. Thus, only verbal consent was required for our study that was approved by the ethics committee (*Universidad Europea de Madrid*, Spain).

### Laboratory tests

Laboratory testing (20 to 24°C, 45 to 55% relative humidity, ∼600 m altitude) was performed before each target race using a conventional protocol including progressive treadmill running (Technogym Run Race 1400 HC, Gambettola, Italy) until volitional exhaustion with continuous heart rate (HR) recording using radio telemetry and a downloadable wristwatch (Accurex Plus, Polar Electro OY, Finland). Heart rate recordings were averaged for every 15 s period. The maximal HR value (HRmax) was computed as the maximum HR value obtained during the tests for every 15 s interval. We also made continuous (‘breath-by-breath) respiratory gas-exchange measurements (Vmax 29 C, Sensormedics, Yorba Linda, Ca, USA) to define each subject's ventilatory (VT) and respiratory compensation threshold (RCT) as detailed elsewhere [37]. We used the HR value associated with the metabolic or ‘intensity’ zones defined by the gas exchange data to define low (Zone 1: HR<HR@VT), medium (Zone 2: HR between HR@VT and HR@RCT) or high intensity (ZONE 3: HR>HR@RCT) zones [14], [19], [37], [38].

### Data recording during races

During the years 2004–2007, data were recorded from a ‘target’ race taking place in a competition season of each individual runner. We have defined a target race as one where the subject trained to attain his best possible performance based on his individual characteristics and training background (e.g., 5 km race for the more middle-distance oriented type of runners and 100 km race for the ultra-endurance runners). Data on pacing was retrieved from the official race protocols published by the organizers of each event. All races were run on certified road courses. All competitions were performed during periods where the environmental conditions were relatively mild (temperature <25°C) and not hypoxic (altitude≤600 m). Heart rate recordings were made every 15 s from the start to the end of each event using the aforementioned radio telemetry system and downloadable wristwatch (Accurex Plus, Polar Electro OY, Finland).

### Data analysis

In order to examine our primary aim, we expressed HR recordings relative to the HRmax (%HRmax) value of each individual observed during their aforementioned maximal laboratory testing. Exercise intensity was also quantified using the three HR zones (1, 2 and 3) previously described. Race distance was expressed in ‘actual’ units (km) or relative to the total distance of each event. Running velocity was expressed in ‘actual units’ (m·s^−1^) or normalized relative to the mean velocity of the event. A detailed explanation of all the variables used in this study is provided in Table 1. Data analysis was primarily accomplished using simple descriptive statistics and data were expressed as mean±SEM).

**Table 1:** Explanations of terms and variables used in the text.

Abbreviation or term	Explanation	Figures in which the terms were used
‘%HRmax’	Percentage heart rate (HR) value *during a race* relative to the *laboratory* maximal (15-s average) heart rate (HRmax) value	Figs. 1 & 3–9.
‘%HRR’	Percentage heart rate (HR) value *during a race* relative to the individual HR reserve (HRR) value. (%HRR sustained during the race = average race HR minus resting HR)×100/(HRmax minus resting HR, where resting HR was the lowest individual waking value recorded within 2 weeks before the target race).	Fig. 4
‘Distance’	Actual distance of each race (in km)	Figs. 1, 2 & 6–8
‘Relative distance’	Proportion of the total race distance in arbitrary units. (Total race distance = 1.0)	Figs. 1 & 2
‘Relative HR normalized’	%HRmax (see above) normalized to the *lowest* (15-s average) HR value achieved during the race. (The latter is given a score of 1.0).	Fig. 1
Relative velocity	Running velocity normalized to the average running velocity (m·s^−1^) during the race. (The latter variable is given a score of 1.0).	Figs. 2 & 6
‘Peak HR’	Peak HR value (15-s average) obtained during the race expressed relative to laboratory HRmax (see above). (The latter variable is given a score of 100%).	Fig. 3
‘Zones 1, 2 and 3’	‘low, medium and high metabolic intensity’, respectively, i.e., race HR values (15-s average data) <HR value @ ventilatory threshold (VT) in previous laboratory testing (Zone 1), between HRVT and HR @ the respiratory compensation threshold (RCT) (zone 2) and >HR@RCT (zone 3)	Fig. 3

To examine the secondary aim of our study, we used a linear regression analysis to assess the pattern of HR response relative to each subject's running ability. To accomplish this we examined the relationship between the average intensity of exercise (expressed as %HRmax) and the duration of each race event (5 km to the marathon). We also reasoned that because HRmax and resting HR in humans is inversely associated with age and fitness level, respectively, we accounted for this potential confounding effect by expressing HR relative to the HR reserve (HRR) of each subject (%HRR). (%HRR sustained during the race = average race HR minus resting HR)×100 / (HRmax minus resting HR, where resting HR was the lowest individual waking value recorded within 2 weeks before the target race). For each regression analysis, we reported the *P* value for the equation and the 95%confidence intervals (95%CI) of the Pearson's correlation coefficient.

For the last aim of our study we examined the *HR response of the event for performance discontinuities* by examining the individual data from observations of abrupt decreases in performance within a given race.

## Results

The various races were completed by a substantial number of athletes: (5 km: n = 42; 10 km: n = 53; half marathon (21.1 km): n = 57; marathon (42.2 km): n = 55; 100 km: n = 4). The mean performance level of the subjects remained stable overall with increasing distance. When expressed relative to the current world record for the event being examined in that event, performance time averaged [mean±SEM (range: min, max)] 64.3±1.8% [47, 92] for 5 km, 68.9±1.4% [44, 86] for 10 km, 64.2±1.1% [45, 83] for the half marathon, 63.1±1.4% [42, 86] for the marathon and 64.8±4.4% [56, 76] for 100 km (N = 4 runners only).

### Primary outcome: HR response and exercise intensity according to race distance

**Figure 1:**
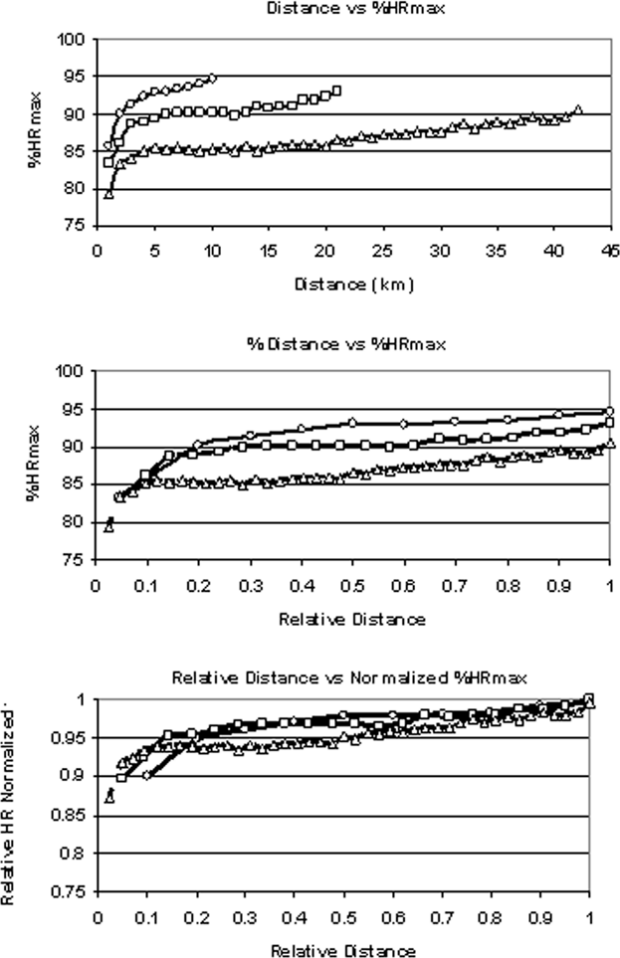
*Top*: Percentage of laboratory maximal heart rate (%HRmax) in relation to distance in races of 10 km (n = 53, symbol = hexagons), 21.1 km (n = 57, symbol = squares) and 42.2 km (n = 55, symbol = triangles). *Middle:* %HR max in relation to the relative distance completed. *Bottom:* %HRmax (normalized to the lower HR achieved during the race, rather than during laboratory testing as in the top and middle figures) in relation to the relative distance completed. Note: For clarity purposes, data are shown as mean (with no SEM).

**Figure 2:**
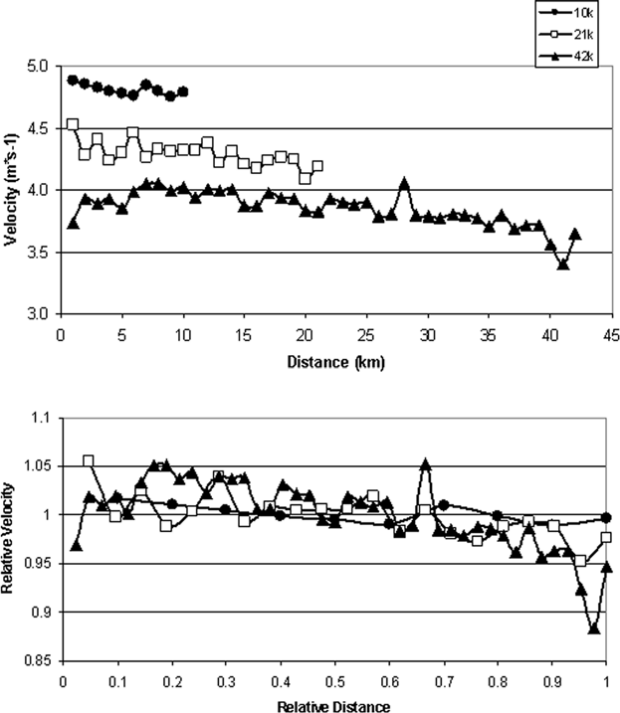
*Top:* Running velocity in relation to the distance completed in 10 km (n = 53), 21.1 km (n = 57) and 42.2 km races (n = 55). *Bottom:* Running velocity normalized to the average running velocity for the event in relation to the relative distance completed. Note: For clarity purposes, data are shown as mean (with no SEM).

As demonstrated in the top panel of figure 1, the HR response expressed relative to the %HRmax during each event was fairly consistent. When the distance was normalized for all distances, the fundamental similarity of the HR response during all three primary race distances became more evident (Fig. 1, middle). When the HR response was normalized for the lower %HRpeak-race (see Table 1 for explanations) achieved during longer races and normalized for distance, the HR response between the events of different durations became essentially constant for all races (Fig. 1, bottom). The pace in these races varied in a characteristic way, whether expressed as actual velocity (Fig. 2, top) or normalized to the average pace during the event which, except for a brief period just before the end of the marathon, remained within ±5% of the average pace (Fig. 2, bottom).

**Figure 3:**
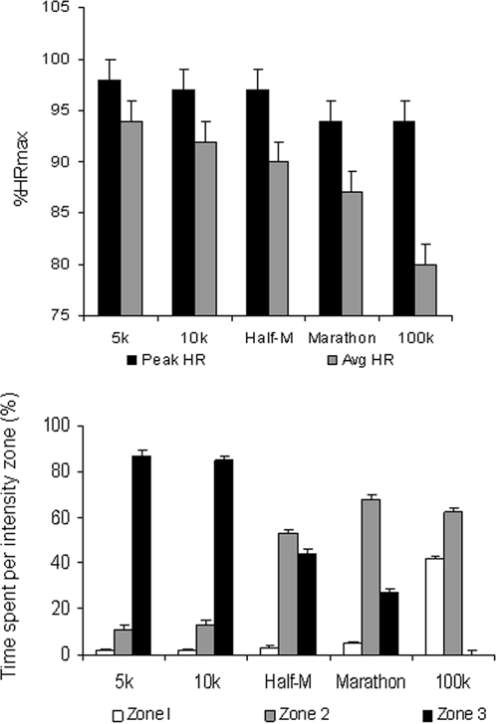
*Top*: Peak and average heart rate (HR) sustained during events of varying durations expressed relative to maximal laboratory heart rate (%HRmax). *Bottom*: Relative proportion of each event with the HR in metabolic or intensity zones defined as <ventilatory threshold (VT) (Zone 1 = low intensity), between VT and the respiratory compensation threshold (RCT) (Zone 2 = moderate intensity), and >RCT (Zone 3 = high intensity). Data are mean±SEM. Abbreviations: Avg (average), Half-M (half marathon, i.e., 21.1 km).

HR and the average HR observed during the event decreased progressively with the duration of the competition (Fig. 3, top). Similarly, the metabolic characteristics of the events changed dramatically with the length of the event. Specifically, HR decreased from high intensity “Zone 3” effort in the 5 and 10 km events (80–85% of total race time) to being virtually absent in the ultra-marathon distances (Figure 3, bottom). Medium intensity effort (‘zone 2’) was predominant in race distances≥half-marathon (40–60% of total running time) but its relative importance was much lower in shorter distances (∼10%). The percentage of ‘low exercise intensity’ (‘zone 1’) was negligible in race distances≤marathon but comparatively very important in 100-km races (∼40% of total running time).

**Figure 4 pone-0002943-g004:**
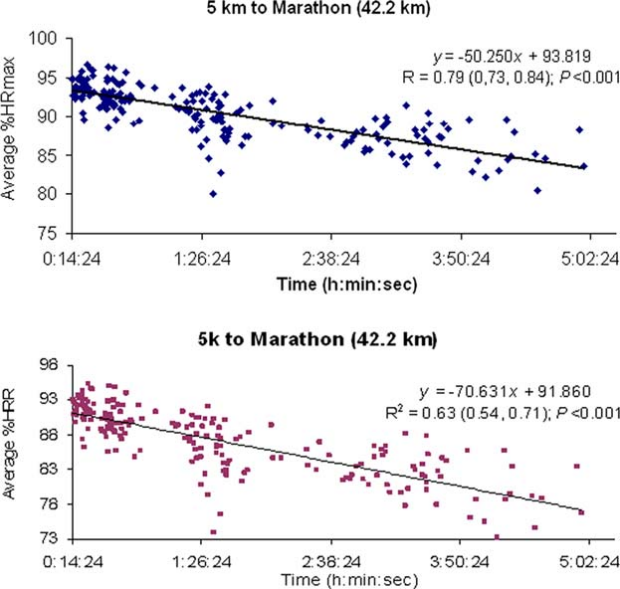
Relationship between average intensity of exercise expressed as average % of maximal heart rate (%HRmax) (*Top*) or as average % of heart rate reserve (%HRR) (*Bottom*) vs. event duration, in races of 5 km to 42.2 km. All individual data points are shown in each Figure and the relation between average %HRmax or %HRR and exercise duration was fitted to a linear regression equation. In the latter, *P* value and Pearson correlation coefficients together with their corresponding 95% confidence intervals are reported.

Finally, the average %HRmax (and thus the average exercise intensity) sustained during the competitions decreased systematically with the duration of the event we examined (Figure 4, top). This result was not influenced by individual differences in subject age or fitness level, as the relationship between average %HRR and event duration was fairly similar to the relationship between %HRmax and even duration (Figure 4, bottom).

### Secondary outcome: HR response according to running ability

**Figure 5 pone-0002943-g005:**
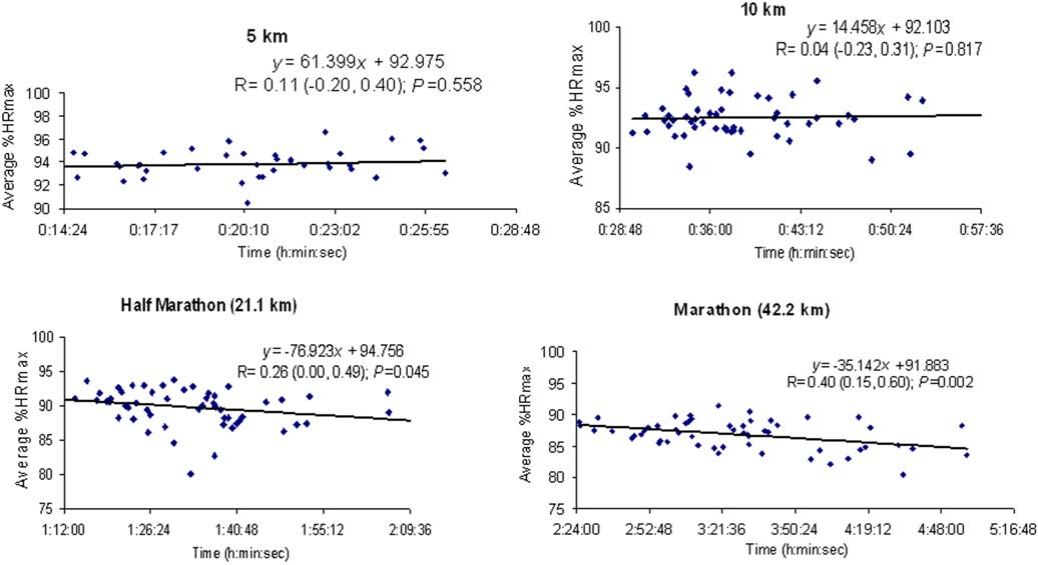
Average intensity of exercise (expressed as average % of maximal heart rate (%HRmax)) within race distances by runners who were relatively faster and relatively slower. All individual data points are shown in each Figure and the relation between average %HRmax and exercise duration in each race was fitted to a linear regression equation. In the latter, *P* value and Peason correlation coefficients together with their corresponding 95% confidence intervals are reported. Overall, the results denote the lack of change in the average %HRmax between the faster vs. the slower runners within a given event, especially up to half-marathon.

There was little evidence that the %HRmax sustained varied with running ability, as the mean %HRmax essentially did not decrease in runners who required more time to complete their events, i.e., we found low Pearson's correlation coefficients for the relationship between mean %HRmax during each event and time to complete each event, especially up to 21 km (Figure 5).

**Figure 6 pone-0002943-g006:**
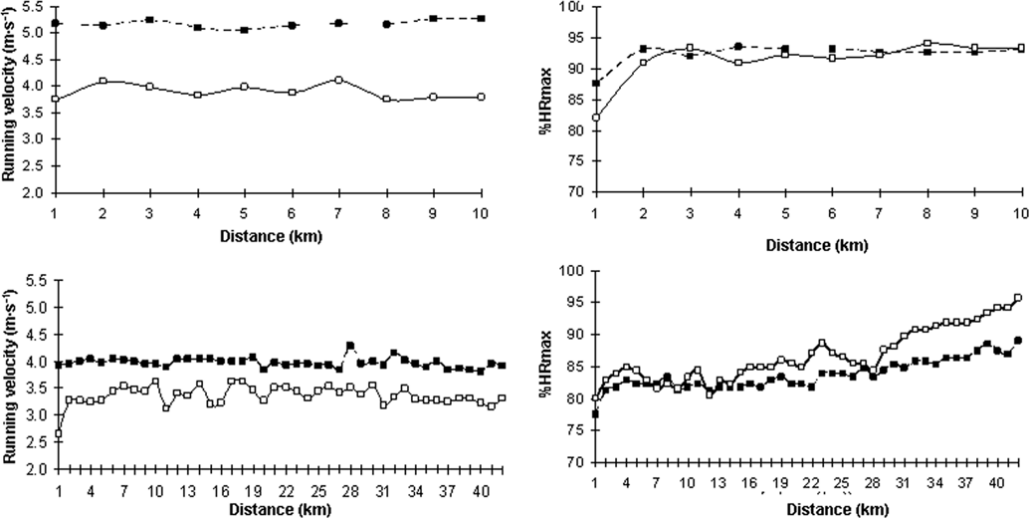
Running velocity (*top/left Figure*) and average intensity of effort (expressed as mean % of maximal heart rate (%HRmax)) for each kilometric check point (*top/right*) in two runners of differing ability during a 10 km race (finishing time of ∼32 (symbol = ▪) vs. ∼43 min (symbol = □); and running velocity (*bottom/left*) and average mean %HRmax for each kilometric check point (*bottom/right*) in two runners of differing ability during a marathon race (finishing time <3 h (▪) vs. >3 h 30 min (□)). Collectively the figures demonstrate that the pattern of %HRmax increase during competition is very similar regardless of running ability.

Examples of the pattern of running velocity and HR in runners of varying ability are presented in Figure 6. In the two upper panels, results from a 10 km event demonstrate very different running velocities between two runners with final times of 43 vs. 32 min. The %HRmax response, however, was essentially identical between the runners. In the two lower panels there was a similar pattern of differences in running velocity and similarity of %HRmax responses in two other runners of different ability in the marathon (>3 h 30 min vs. <3 h).

### Tertiary outcome: HR response in the event of performance discontinuity

**Figure 7 pone-0002943-g007:**
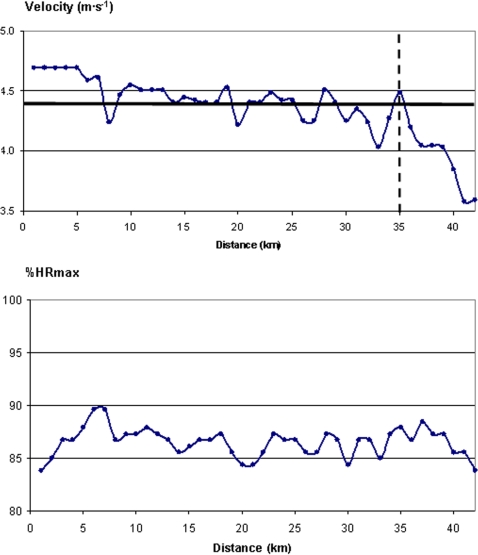
Running velocity (*top*) and % of maximal heart rate (%HRmax) (*bottom*) in a marathon runner who “hit the wall” (vertical dashed line) and decreased running velocity markedly after 35 km. Mean speed during the race is marked with a bold horizontal line. Note that %HRmax did not decrease until after a secondary decrease in pace at 39 km, suggesting that despite slowing after 35 km, he maintained effort until slowing down even more at 39 km.

The pattern of running velocity and HR in runners with discontinuities in performance is presented in Figures 7–9. In Figure 7 we present the results of a runner who “hit the wall” in the marathon. ‘The wall’ refers to the point, generally at ≥32 km, where glycogen stores are depleted and thus energy for skeletal muscle contraction comes mainly from fat oxidation. This point distinguishes a physiologic “shift,” representing a comparatively slower metabolic process than glycogenolysis resulting in i) decreased muscle output and ii) shortage of glucose to the brain with subsequent hypoglycaemia. Clinically, ‘the wall’ is characterized by several unpleasant symptoms such as a lack of physical coordination, paraesthesia in toes and fingers, nausea, muscle spasms, dizziness, inability to think clearly, and extreme physical weakness [39]. In our current example, the athlete was running at a pace that would have allowed him to finish in <2 h 40 min before reaching the 35th km of the race. Between 35–39 km of the race his pace slowed to 93% of his intended value. After 39 km he slowed further to 81% of the pace recorded in the first 35 km. On this basis, the runner lost ∼5 min during the last 7 km compared to the pace he had been sustaining over the first 35 km. His %HRmax, however, did not decrease until after the secondary deceleration after 39 km. This presumably suggests that the runner was maintaining his effort despite the loss of muscle power output.

**Figure 8 pone-0002943-g008:**
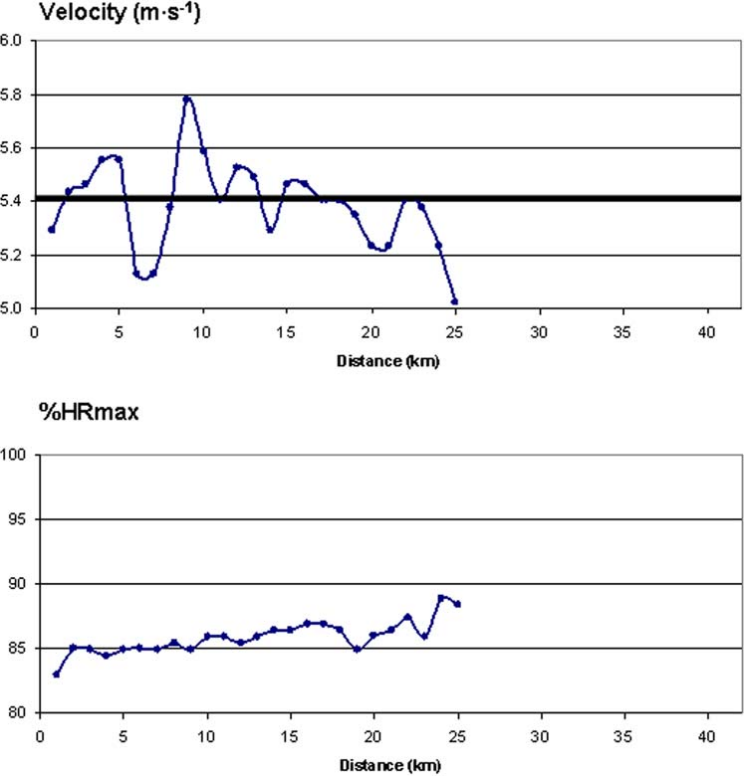
Running velocity (*top*) and % of maximal heart rate (%HRmax) (*bottom*) in an experienced marathon runner (best performance = 2 h 15 min) who was attempting to run at 2 h 10 min pace (which corresponds to the mean speed marked with a bold horizontal line in the upper Figure) until he had to drop out at 25 km. Note that his %HRmax continued to increase until he dropped out of the race, indicating that despite slowing his running velocity, his relative effort was still increasing throughout the run.

**Figure 9:**
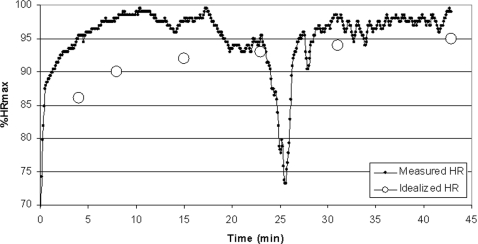
Intensity (expressed as % of maximal heart rate, %HRmax) versus time curve of an elite runner who started a 12-km cross-country race too fast, slowed to a walk in mid race, and then continued the race. It is contrasted to an idealized curve for this distance (based on the data in Figure 1). Abbreviations: HR (heart rate).

In Figure 8 we present by comparison the results of an elite East-African runner who was not a part of our primary group of runners, but whose best performance time in the marathon was 2 h 15 min during a major marathon race. Over the first 22 km he ran at a pace that would have resulted in a time of 2 h 10 min. At this point he became fatigued, slowed his running pace despite continuing effort (as evidenced by the increasing %HRmax) and dropped out of the race at 25 km. In Figure 9 are the results of another elite runner (not part of the primary group of runners studied, best performance time in marathon: 2 h 16 min) who started a 12-km cross country race too ambitiously. He led for the first 4 km against runners who were better at this distance, progressively slowed over the next 3 km, stopped to walk briefly at 7 km, and then finished running, but in a much worse time than expected. When his %HRmax versus time curve is compared to what might be expected for a race of this distance as presented in Figure 1, it is clear that he was working much harder early in the race than ideal. After slowing to recover, his %HRmax was fairly close to what might be expected from an idealized race. At the point that he stopped to walk (essentially a ‘competitive catastrophe’) he could just as easily have dropped out of the race, as did the runner depicted in Figure 8.

## Discussion

In the development of our investigation, we proposed three primary hypotheses related to running performance in humans. Underlying each hypothesis we proposed that various physiological systems interact to preserve homeostasis for the period of time associated with a running event in an attempt to regulate running effort [1]. Based on our findings, we believe that several outcomes add to current body of knowledge regarding running intensity and pacing during competitive circumstances. In light of these findings, we also offer several limitations to our study at the end of our discussion.

### Hypothesis 1: HR as a marker of exercise intensity -Influence of race distance

Our results confirm our first hypothesis that HR increases in a consistent pattern during competitive events and appears to be scaled proportionally to the distance of the event. When the highest (‘peak’) HR observed during individual races was used as a method of scaling the progression in intensity of physical effort, it appears that the normalized intensity grows in concert with the relative distance. This finding is consistent with evidence for a scaled growth of fatigue during competition ranging from only a few minutes to several weeks and implies that the degree of physiologic strain is controlled in an active way [9], [10], [19], [27], [28].

Evidence that running pace varies to allow a controlled growth of relative strain, as reflected by the %HRmax response, further supports the concept that athletes are continually in a dialogue or negotiation with themselves, assessing how fatigued they feel [40]. Especially, peripheral muscle fatigue would be the highly, constantly regulated variable [32], with a continuous sensory feed-back coming from working muscles to the CNS so as to ensure that muscle fatigue is confined to a certain level or ‘threshold’ [33], above which potentially dangerous consequences, especially muscle structural damage, could occur [34].

### Hypothesis 2: Adept vs. non-adept runners

For our second hypothesis, we theorized that adept and less adept runners would display similar HR responses during competition despite different running speed relative to their individual ability. We further proposed that the relative physiologic strain would be proportional to the duration of effort. We developed this hypothesis based on the common belief that better athletes can ‘dig deeper’ and work relatively harder than their less successful counterparts. However, evidence from this study does not support this concept.

In our current study, we found that the pattern of %HRmax response during an event was very similar in all athletes despite a wide variety of competition abilities and large differences in running performance. This evidence suggests that adept runners are faster due of their underlying physiological capacity rather than because they put more relative effort into their competition.

### Hypothesis 3: HR response during discontinuities in performance

The third hypothesis of our study stated that instances involving a discontinuity in performance are related to poor regulation of the physiologic strain. Our data confirm our hypothesis as we found evidence that in the case where there is a discontinuity in running performance (i.e., muscle task failure), it is associated with a pattern of increased effort, as the athlete tries to maintain effort in the face of gross failures in muscle output. This argues against the concept of Noakes et al. [26] and St Clair Gibson and Noakes [24] who have hypothesized that fatigue and reductions in muscle power output are evidence of an active reduction in effort (i.e., reduced neural output to working muscles) due to a pre-existing template or plan for an event which is based on either practice or prior competitive experience.

The continual maintenance of %HRmax even after the athlete begins to slow suggests that the effort is not ‘turned off’ immediately. Further, there are enough examples of what might be termed ‘physiological catastrophes’ or those events where an athlete experiences a dramatic loss in body homeostasis that can potentially result into life-threatening collapses. Classic examples of such events during high level competition include Dorando Pietri's collapse at the London Olympics in 1908, the collapse of Jim Peters in the marathon of the 1954 Empire Games held in Vancouver, the collapse and death of Tom Simpson on Mont Ventoux during the 1967 Tour de France, the deaths during the cycling races during the 1960 Olympics in Rome, the dramatic staggering finish of Gabriela Anderson-Schiess in the woman's marathon at the 1984 Olympics in Los Angeles and the non-trivial number of deaths during competition (particularly from heat stroke). These examples support the idea that ‘physiological catastrophes’ can and do occur with some frequency during competition precisely because the athletes were either unwilling or unable to down-regulate effort despite dangerously high levels of strain. In the event of a pre-existing (frequently undiagnosed) cardiac disorder [41] or drug abuse, as in the case of Tom Simpson [15], the inability to regulate effort or over-ride a “central governor” can be fatal even in well accomplished athletes.

### Methodological limitations

We aware that the main methodological limitation of using HR recordings to monitor physiological strain is due to the phenomenon known as “cardiac drift” [42]. Cardiac drift is characterized by a gradual increase in HR values that tends to occur during prolonged exercise involving large muscle mass (e.g., running) despite maintaining “*external* load” (e.g., running velocity) and which does not solely reflect an increase in actual physiological intensity (“*internal* load”). This phenomenon is most marked in hot environments. (In this regard, environmental conditions were relatively benign in our study, with temperature consistently <25°C, as detailed in the Methods section). Further, the magnitude of the HR drift phenomenon is expected to increase with exercise duration. As such, it would be higher in the slowest compared to the fastest runners within a given long distance race (i.e., half marathon and above), and thus would artificially inflate exercise intensity in the former.

Though we acknowledge this “limitation,” we are unaware of any other index capable of assessing physiologic strain during competitive running without significantly interfering with the running event. Further, the gradual increase in HR that inevitably occurs during a given endurance exercise bout despite maintaining constant *external* load mimics, at least partly, the gradual increase in VO_2_ (and, as such, the actual exercise intensity or physiological *internal* load) that is known to occur during endurance exercise bouts at constant *external* loads –that is, the so-called oxygen uptake (VO_2_) slow component phenomenon [43]. The VO_2_ slow component occurs mainly at moderate-to-high intensities (i.e., >VT or zones 2–3) [43]. In this regard, it must be kept in mind that, except in 100 km races, the predominant exercise intensity of most events studied in this report corresponded to zones 2–3 (Figure 3).

An additional limitation arises from the fact that, for simplicity purposes, we did not use the original TRIMP (*training impulse*) model [43], [44] or its more recent modified version [19], [37], [38] to integrate total exercise loads (metabolic intensity×total exercise time) into a single algorithm. We thus propose that future research in the field use the TRIMP model as well as other possible variables (e.g., VO_2max_, running velocity at the VT and RCT) to evaluate the growth of *internal* (physiologic strain) and *external* loads during actual endurance running competitions ranging from less than one hour (5–10 km) to several hours (42 km and above).

In summary, HR, a key index of endurance exercise intensity and thus of an athlete's effort increases in a consistent pattern during competitive events that is proportional to the event distance. Since the sustained relative effort is the same irrespective of an athlete's competition level, the better performance of elite runners is simply attributable to their superior physiological capacity. When there is a significant reduction in running performance, particularly, a dramatic drop-off in speed during the last part of a race, it is often associated with evidence of increased effort in the face of failures to maintaining muscular power output. This would explain the not-infrequent occurrence of collapses evident in the history of endurance competitions.
